# Medição do Fluxo Sanguíneo Coronariano por Angiocoronariografia Convencional por um Novo Método Baseado na Detecção da Densidade de Contraste

**DOI:** 10.36660/abc.20200936

**Published:** 2020-09-18

**Authors:** Hector M. Garcia-Garcia, Pablo Blanco

**Affiliations:** 1 MedStar Washington Hospital Center Departamento de Cardiologia Intervencionista Washington DC EUA MedStar Washington Hospital Center – Departamento de Cardiologia Intervencionista, Washington, DC - EUA; 2 Georgetown University School of Medicine Washington DC EUA Georgetown University School of Medicine, Washington, DC - EUA; 3 Laboratório Nacional de Computação Científica Petrópolis RJ Brasil Laboratório Nacional de Computação Científica, Petrópolis, RJ - Brasil; 4 Instituto Nacional de Ciência e Tecnologia em Medicina Assistida por Computação Científica Petrópolis RJ Brasil Instituto Nacional de Ciência e Tecnologia em Medicina Assistida por Computação Científica,Petrópolis, RJ - Brasil

**Keywords:** Fluxo Sanguíneo Regional, Pulso Arterial, Circulação Coronária, Sensibilidade de Contraste, Angiografia Coronária/métodos

Desde os primórdios de sua utilização, a avaliação do fluxo coronariano indireto por angiocoronariografia (ACG) tem sido relatada em todas as notas laboratoriais de hemodinâmica, pois é considerada uma ferramenta prognóstica, particularmente no contexto da intervenção percutânea coronariana primária. A trombólise no sistema de grau de fluxo do infarto do miocárdio (TIMI) e, com menos frequência, na contagem de quadros TIMI corrigida (*corrected TIMI Frame Count — CTFC*) são os dois escores mais comumente usados para refletir o estado do fluxo sanguíneo coronariano (FSC).^1^ Nesta edição, Lopez-Hidalgo e Eblen-Zajjur relatam^2^ uma nova abordagem de medição angiográfica quantitativa do FSC com base na detecção de contraste densitométrico (DM) em ACG offline. Para tanto, trinta pacientes foram estudados e divididos em 2 grupos: grupo de fluxo sanguíneo coronariano normal (FN) e fluxo sanguíneo coronariano lento (FL), de acordo com a CTFC. Os autores concluíram que o novo método DM era viável e demonstrava a capacidade de diferenciar fluxo normal (FN) e fluxo lento (FL) em pacientes com dor torácica e artérias coronarianas normais.

Os autores desenvolveram uma abordagem criativa para medir o FSC quantitativamente e objetivamente, de forma indireta. Essa abordagem supera parcialmente as limitações dos escores tradicionais TIMI e CTFC. Por este motivo, gostaríamos de parabenizar os autores por este relatório inovador. Isto posto, gostaríamos de destacar alguns pontos que merecem uma discussão mais aprofundada. Primeiro, houve uma tentativa de definir fluxo coronariano “normal”. Com base na população estudada, derivou-se o ponto de corte da ACG. Nunca se pode ter certeza se esses pacientes têm realmente FSC normal. Tradicionalmente, os métodos não invasivos (como tomografia por emissão de pósitrons e ressonância magnética cardíaca) e métodos invasivos (ou seja, guia-Doppler e termodiluição) são considerados as melhores abordagens para medir o FSC e, para cada tecnologia, são conhecidos os valores padrão de normalidade.^3^ Assim, no relatório, a definição de valores “normais” pode ser, na melhor das hipóteses, um substituto aproximado do FSC real.

Em segundo lugar, o uso de uma abordagem de DM que requeira o uso de um software offline pode complicar a adoção dessa ferramenta para fins clínicos. Em terceiro lugar, as medições são realizadas na fase de *washout* para a qual não há uma definição clara da janela de tempo, podendo ser influenciadas pelo tipo de contraste usado, modo de injeção de contraste (manual vs. automático), duração da filmagem e, possivelmente, pela injeção de nitratos intracoronários, que em alguns laboratórios de hemodinâmica são administrados rotineiramente, enquanto em outros podem não ser injetados. Além disso, apenas intervalos de quartil subjetivamente menores foram fornecidos como evidência para o uso da fase de *washout*. Nenhuma evidência estatisticamente significativa foi fornecida.

Aplaudimos qualquer esforço para tornar nossas avaliações de ACG mais quantitativas e objetivas, o que pode ser feito usando a abordagem densitométrica apresentada neste relatório, mas antes que alguém possa considerar seu uso clínico, seria necessária uma validação extensa.
