# Supplementary material for: Medição do Fluxo Sanguíneo Coronariano por Angiocoronariografia Convencional por um Novo Método Baseado na Detecção da Densidade de Contraste
Source: Arq Bras Cardiol. 2020 Sep 18;115(3):513–4. [Article in Portuguese] doi: 10.36660/abc.20200936 (PMC9363098; doi:10.36660/abc.20200936)
Supplement: Translation

# Translation: Coronary Blood Flow Measurement in Conventional Coronary Angiograms by a New Method Based on Contrast Density Detection

**Authors:** Hector M. Garcia-Garcia, Pablo Blanco

**Affiliations:** 1 USA MedStar Washington Hospital Center – Departamento de Cardiologia Intervencionista, Washington, DC - USA; 2 USA Georgetown University School of Medicine, Washington, DC - USA; 3 Brazil Laboratório Nacional de Computação Científica, Petrópolis, RJ - Brazil; 4 Brazil Instituto Nacional de Ciência e Tecnologia em Medicina Assistida por Computação Científica, Petrópolis, RJ - Brazil

**Keywords:** Regional Blood Flow Pulse, Coronary Circulation, Contrast Sensitivity, Coronary Angiography/methods

From its inception, indirect coronary flow assessment by coronary angiography (CAG) has been reported in every catheterization laboratory note as it is regarded a prognostic tool particularly in the context of primary percutaneous coronary intervention. Thrombolysis in myocardial infarction (TIMI) flow grade system and, somewhat less often, corrected TIMI Frame Count (CTFC) are the two most commonly used scores to reflect the status of coronary blood flow (CBF).^1^ In this issue, Lopez-Hidalgo and Eblen-Zajjur report^2^ a new approach of quantitative angiographic measurement of CBF based on densitometric (DM) contrast detection in off-line CAG. To this aim, thirty patients were studied and divided into 2 groups: normal coronary blood flow (NF) and slow coronary blood flow (SF), according to the CTFC. The authors concluded that the new DM method was feasible and showed some capability to differentiate between normal flow (NF) and slow flow (SF) in patients with chest pain and normal coronary arteries.

The authors have devised a creative approach to indirect measurement of CBF, both quantitatively and objectively. This approach partly overcomes the limitations of the traditional TIMI and CTFC scores. For this reason, we would like to congratulate the authors on this novel report. Having said this, some points deserve further discussion. First, there was an attempt to define “normal” coronary flow. Using the study population, the cut-off was derived from CAG. One can never be sure whether these patients have actually normal CBF. Traditionally, non-invasive methods (such as Positron Emission Tomography and Cardiac Magnetic Resonance Imaging) and invasive methods (i.e. Doppler Wire and thermodilution) are considered the best approaches to measure CBF and standard normality values are known for each technology.^3^ In the report, the definition of “normal” values may be, at best, an approximated surrogate to the real CBF.

Secondly, using a DM approach which requires an off-line software might complicate the adoption of this tool for clinical purposes. Thirdly, the measurements are performed in the washout phase for which there is not a clear definition of such a time window, and might be influenced by the type of contrast used, mode of contrast injection (manual vs. automatic), the filming length and potentially by the injection of intracoronary nitrates which, in some cath labs, are administered routinely while in others it may not be injected at all. In addition, only subjectively narrower quartile ranges were provided as evidence for using the washout phase and no statistically significant evidence was given.

We applaud any effort to make our CAG assessments more quantitative and objective, which can be achieved by using the densitometric approach presented in this report, but before considering using it clinically, extensive validation would be required.
